# Percutaneous screws CT guided to fix sacroiliac joint in tile C pelvic injury. Outcomes at 5 years of follow-up

**DOI:** 10.1051/sicotj/2018047

**Published:** 2018-11-27

**Authors:** Gabriele Falzarano, Giuseppe Rollo, Michele Bisaccia, Valerio Pace, Riccardo Maria Lanzetti, Esteban Garcia-Prieto, Paolo Pichierri, Luigi Meccariello

**Affiliations:** 1 Department of Orthopedics and Traumatology, Azienda Ospedaliera “Gaetano Rummo”, Benevento Italy; 2 Department of Orthopedics and Traumatology, Vito Fazzi Hospital, Lecce Italy; 3 Division of Orthopedics and Trauma Surgery, University of Perugia, “S. Maria della Misericordia” Hospital, Perugia Italy; 4 Department of Trauma and Orthopaedics, The Royal National Orthopaedic Hospital, Stanmore, London UK; 5 Department of Orthopaedics, “Hospital General de Villalba”, 28400 Collado Villalba Spain

**Keywords:** Tile C fractures, Percutaneous osteosynthesis, Sacroiliac injuries, Cannulated screws, Pelvic injury.

## Abstract

*Introduction:* The treatment of the sacroiliac joint (SIJ) vertical instability is a matter of current discussions and remains controversial. The aim of our study is the evaluation of the surgical management of SIJ vertical instability involving the use of cannulated screws introduced under CT guidance and local anesthesia.

*Materials and methods*: In the set time frame of 7 years, 96 poly-trauma patients with Tile's type C fracture of the pelvis with vertical instability of the SIJ were treated. The average distance between the two stumps was 73.4 mm (range: 43–100 mm). All patients were treated with anterior stabilization and subsequent stabilization with cannulated screws (Asnis^®^ Stryker^®^ 6 mm, an average length of 70 mm; range from 55 to 85 mm) of the sacroiliac fracture. The clinical and radiological follow-up was performed with follow-up plain radiograph and Majeed score (from 1 to 60 months after injury).

*Results*: The consolidation of pelvic fractures was obtained after an average of 63 days. The average Majeed score was as follows: 96 points at 1 month, 84 points at 3 months, 62 points at 6 months, 44 points at 12 months, 42 points at 24 months, 32 points at 36 months, 28 points at 48 months and 28 points at 60 months. Complications were as follows: not fatal deep vein thrombosis in five cases, skin infection at the entry point of the screws in six cases, screw breakage in one case and loosening of the screws in one case. Radiological evidence of fracture consolidation was achieved on average at 63 days. Forty-seven patients managed to get back to their pre-trauma employment at the end of the convalescence period.

*Conclusions*: Our results suggest that the stabilization of SI Tile type C fracture/dislocations with CT-guided percutaneous cannulated screws is a valid and feasible management option and associated with a low complication rate.

## Introduction

Sacroiliac (SI) instability may result from the disruption of the sacroiliac joint (sacroiliac dislocation) or a fracture/dislocation of the ilium (trans-iliac) or the sacrum (trans-sacral). The mechanisms of injury may include combination of axial load and abrupt rotation; ligament, capsular or synovial disruption or tension; shearing forces; abnormal joint mechanics; pathological changes [1].

Pelvic fractures are classified by using the Tile classification [2]. This divides pelvic fractures into three groups (each divided in further three subgroups): type A (stable − posterior arch is intact), type B (rotationally unstable, vertically stable − incomplete disruption of the posterior arch), type C (rotationally and vertically unstable − complete disruption of the posterior arch).

The lesions of the sacroiliac joints and sacroiliac fractures may be associated with partial or complete tear of the posterior ligamentous complex, resulting in a rotational and vertical instability of the pelvic ring [3]. The above-mentioned unstable or displaced lesions found in Tile type C fractures must be anatomically reduced and stabilized by internal fixation. Chronic instability and consequent permanent disability may be caused in case of inadequate treatment of these injuries [4–9].

Sacroiliac fracture stabilization with cannulated screw can be an elegant, clever and relevant method to stabilize such lesions. Lambotte already described this technique in 1913 [10]. In 1978, Letourel described the treatment of sacroiliac instability with cannulated screws with patients in prone position under radiographic control [10]. Other authors as Matta and Routt have shown how efficient the use of cannulated screws is in reducing complication rates even with surgery performed with patients in supine position. Traditional open surgery reduction procedures may often show limits connected with complications and high infection rate [4–9].

Furthermore, the literature shows that the use of cannulated screws for Tile C pelvic fractures is an appropriate management option which guarantees at least satisfactory (and usually excellent) clinical results. We have therefore adopted this management strategy for our trauma inpatients diagnosed with such type of fracture requiring surgical treatment.

In this study, we describe our experience in treating surgically 96 patients after sustaining vertically unstable Tile type C pelvic fractures using CT-guided percutaneous cannulated screws. Our aim is to present our results and compare them to the international literature with the scope to confirm the appropriateness of our management of vertically unstable Tile type C pelvic fractures and his good results.

## Materials and methods

In the set time frame of 7 years, we treated 96 patients (see Table 1) after sustaining Tile C type pelvic fractures with sacroiliac vertical instability (with clinical and radiological evidence after anteroposterior, inlet and outlet pelvic plain radiographs) in the same trauma unit.

**Table 1:** Description of population.

Number of patients	96
Average age of patients in years old (range)	37.3 years old (range: 19–63 years old)
Number of males and number of females	86 male; 10 female
Type of pelvic injury according to Tiles Classification	Type C of tile
Type of trauma	40 car accident, 20 motor bike accident, 16 farm vehicle
Number of internal organs injured	24 broken spleens, 30 liver contusions, 48 brain contusions, 22 injured urethras, 58 pneumothoraxes
Number of associated skeletal fractures	14 fractured wrists (2 bilateral case), 20 diaphyseal fractures of the femur, 14 tibia fractures (8 open fractures), 4 tibial plateaux fractures, 8 forearm fractures, 2 elbow dislocations, 2 fractures of the C4 vertebral body (intact spinal cord)
Average displacement of sacroiliac joint after trauma in mm (range)	73.4 mm (range: 43–100 mm)
Number of embolized patients	18
Time to trauma to surgery in days (range)	7.4 days (range: 3–16 days)
Length of stay of patients in intensive care after operation in day (range)	4.7 days (1–15 days)
Type of screws used for fixation	Asnis^®^(Stryker^®^) 6 mm
Average length of the screws in mm (range)	70 mm (range: 55–85 mm)
Used imaging to drive to insert the screws	CT
Follow-up in months (range)	60 months (range: 60–98 months)

The mean age was 37.3 (range: 19–63 years). Eighty-six patients were males and 10 were females. All injuries resulted from high-speed road accidents (40 car, 20 motorcycle, 16 agricultural vehicles).

Pelvic fractures were classified as Tile C1 fracture in 51 patients, Tile C2 in 28 and fracture in 23 patients.

In all 72 patients had associated injuries: internal lesions (24 ruptures of the spleen, 30 liver contusions, 48 head trauma, 22 urethra lesions, 58 pneumothoraces), 24 associated fractures (14 distal radial epiphyses fractures and in 2 cases bilateral fractures, 20 femoral shaft fractures, 14 tibial fractures 8 of which were compound, 4 tibial plateaux fractures, 8 forearm fractures, 2 elbow fracture/dislocations, 2 C4 stable fractures).Eighteen cases required embolization due to concomitant arterial injury causing hemodynamic instability. No mortality was recorded.

Patients were medically fitted to be surgically treated with the studied procedure (no active bleeding, hemodynamically stable, life and limb threatening injuries treated, agreement of the anesthetic team with completion of preoperative routine blood tests and investigations, medical and surgical conditions able to put patients at inappropriate threat with risks outweighing benefits treated before performing the studied procedure).

**Figure 1:**
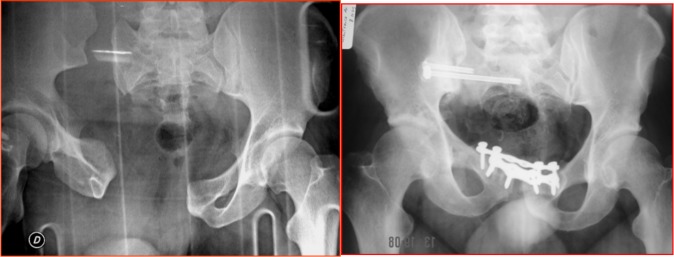
Case of a female patient (42 years old) with right sacroiliac joint instability (C1 Tile injury) and opening of the pubic symphysis following suicidal attempt by fall from height (picture on the left). The patient was initially treated with reduction of the vertical shear and double plate fixation of the pubic symphysis using (anterior approach) and this was followed after 3 days by percutaneous CT-guided screw fixation of the sacroiliac joint (picture on the right).

**Figure 2:**
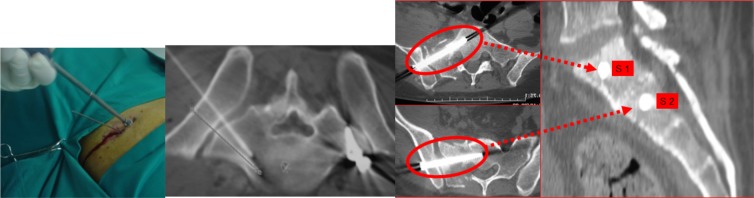
Case of a male patient (46 years old) who sustained a B2 Tile fracture injury with a Dennis III fracture of the iliac wing following a car accident. The first picture from the left shows the positioning of a percutaneous screw. The other three pictures (two coronal and one sagittal view of intra-op CT scan) show the appropriate screw positioning within S1 and S2 vertebral body with preservation of the perisacral neurovascular structures.

The 96 patients were treated surgically 7.4 days on average after admission (range: 3–16 days). First, different techniques of anterior pelvic stabilization were performed. Then, CT-guided percutaneous cannulated screws were used for the posterior stabilization in all cases (Figure 1). Patients were positioned on CT table with the affected side up. The entry points on the skin and correct angles were initially searched by the obtainment of axial CT images. Entry points were then marked on the skin. This was followed by draping and prepping of the sterile field. Laser guide was used to further confirm the entry points. Guide pins were inserted at pre-set angles through the skin after making skin incision of about 1 cm in length. Pins were pushed in till achievement of the outer cortex of the lateral wall of ilium under accurate CT guidance (Figure 2). CT images were used to confirm the made route for the screws and their appropriateness and check absence of violation of neurovascular structures and sacral foramina. Guide wires were used to insert the cannulated screws, whose length was checked and determined using images obtained with the CT scanner. The cannulated screws were then inserted (S1 and S2 level). The CT scanner was used again to confirm the correct position of the screws.

The average distance between the two stumps was 73.4 mm (range: 43–100 mm). About 6 mm Asnis^®^ (Stryker^®^) cannulated screws with an average length of 70 mm (range: 55—85 mm range) were used for all patients.

All patients were discharged with anti-thromboembolic prophylaxis with enoxaparin 50 IU/KG (International Units/kilogram) per day and serial echo-Doppler controls at 1–3 months [24–28]. Patients were allowed to weight bear on their affected limbs with partial load from the 65th (range: 60–75 days) postoperative day (on average), reaching a total weight bearing on their limbs around the 96th postoperative day (range: 86–108 days).

The following follow-up program was organized for all included patients: clinical evaluation, radiographic control with pelvic projections (Antero-Posterior view, inlet and outlet views), Majeed score [23], record of return to work timing and recorded complications at 1 month, 3 months, 6 months, 12 months, 24 months, 36 months, 48 months and 60 months.

The fifth year was chosen as the cut-off follow-up period of the study to homogenize the group and have a common end point. In fact, there are still currently patients being reviewed in their eighth year of follow-up. Patients' notes and electronic hospital records were also used to perform our data collection. Data were recorded using Microsoft Excel and Word. This was followed by data analysis with statistical calculation, which included descriptive statistics used to summarize the characteristics of the studied group. Mean ages of the patients were rounded to the closest year. Mean Majeed scores and days of admissions before undergoing surgical management were also calculated. Statistical analyses were performed with SPSS v.15.0 (SPSS Inc., an IBM Company, Chicago, IL, USA).

The study complies with the Declaration of Helsinki in 1964, amended in 2000, and none of the authors had received direct or indirect compensation for the realization of this study. All patients provided informed consent for the participation in the study.

## Results

All 96 patients underwent the described and studied surgical procedure. The average length of stay was 15 days (range: 12–33 days). No significant medical complication requiring admission to the intensive care unit was recorded.

The Majeed score (see Table 2) was on average: 96 points at 1 month (range: 94–100 points), 84 points at 3 months (range: 78–92 points), 62 points at 6 months (range: 56–78 points), 44 points at 12 months (range: 34–64 points), 42 points at 24 months (range: 28–64 points), 32 points at 36 months (range: 24–54 points), 28 points at 48 months (range: 24–54 points), 28 points at 60 months (range: 24–54 points).

**Table 2:** Trends in the population of the Majeed score during the 60-month follow-up.

Time in months after the surgery	Majeed score in points (range)
1 month	96 (94–100 points)
3 months	84 (78–92 points)
6 months	62 (56–78 points)
12 months	44 (34–64 points)
24 months	42(28–64 points)
36 months	32 (24–54 points)
48 months	28 (24–54 points)
60 months	28 (24–54 points)
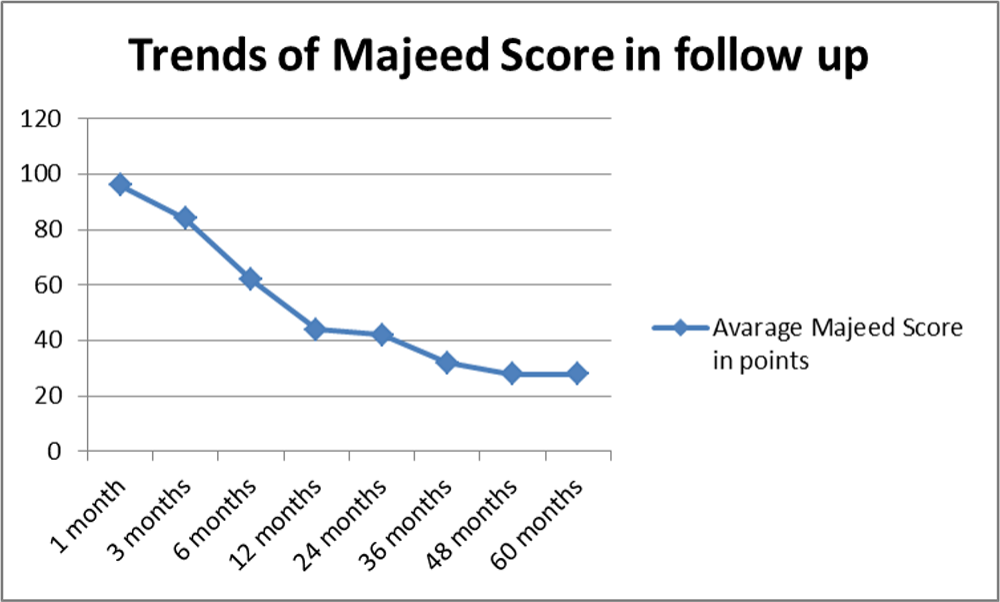	

The Majeed score significantly and gradually improved throughout the entire follow-up reviews. The most significant improvement was recorded during the first post-op year (from 96 points at 1 month post-op down to 44 points at 12 months). Another significant improvement was noted between 24 months and 36 months of follow-up. Stable results were recorded at 4 and 5 years post-op.

Implants in 93 patients appeared stable at radiographic control with no signs of loosening or mal-positioning. We recorded two cases of metalwork mobilization diagnosed at 16 months (which were successfully surgically treated with removal of metalwork procedures without further complications) and one case of metalwork breakage in a psychiatric patient (see Table 3). None of the patients (neither the above described complicated cases) had documented neurological injuries.

**Table 3:** Number and type of complications during the 60 months of follow-up.

Type of complications	Number of patients
Mobilization of the screws in Sacroiliac Joint	Two at 16 months after surgery
Broken screws	One in psychiatric patient
Nonunion	0
Deep vein thrombosis (%)	11.3%
Superficial infection of the screw wounds(%)	12.5%
Number of patients who returned to the same job before the trauma (%)	51%

Radiological evidence of fracture consolidation of the pelvic fractures was achieved on average at 63 days (range: 56–73 days). No screw mal-positioning cases were recorded following review of plain radiographs in the postoperative period and at the setup follow-ups.

About 11.3% of the patients developed non-clinically significant deep vein thrombosis within the first 3 months post-op, despite anti-thromboembolic prophylaxis. These were diagnosed by performing lower limb echo-Doppler scans (see Table 3) following onset of symptoms and clinical evaluation.

About 12.5% of the patients developed superficial soft tissue infections at point of entry of the screws. About 6.5% of the patients developed superficial soft tissue infections at the level of the surgical wound made for the anterior stabilization of the fracture. All these cases were successfully treated with oral antibiotic therapy (see Table 3).

No metalwork breakage or mal-positioning was recorded with regard to anterior stabilization procedures. Blood transfusion was needed in 82.2% of the patients following anterior stabilization.

Short- and long-term complications and outcomes with regard to the associated injuries (long bone fractures, spleen injuries, etc.) were not recorded.

Forty-seven patients (about 49% of the total) managed to get back to their pre-trauma employment at the end of the convalescence period; the remaining 51% had to change type of job.

## Discussion

**Figure 3:**
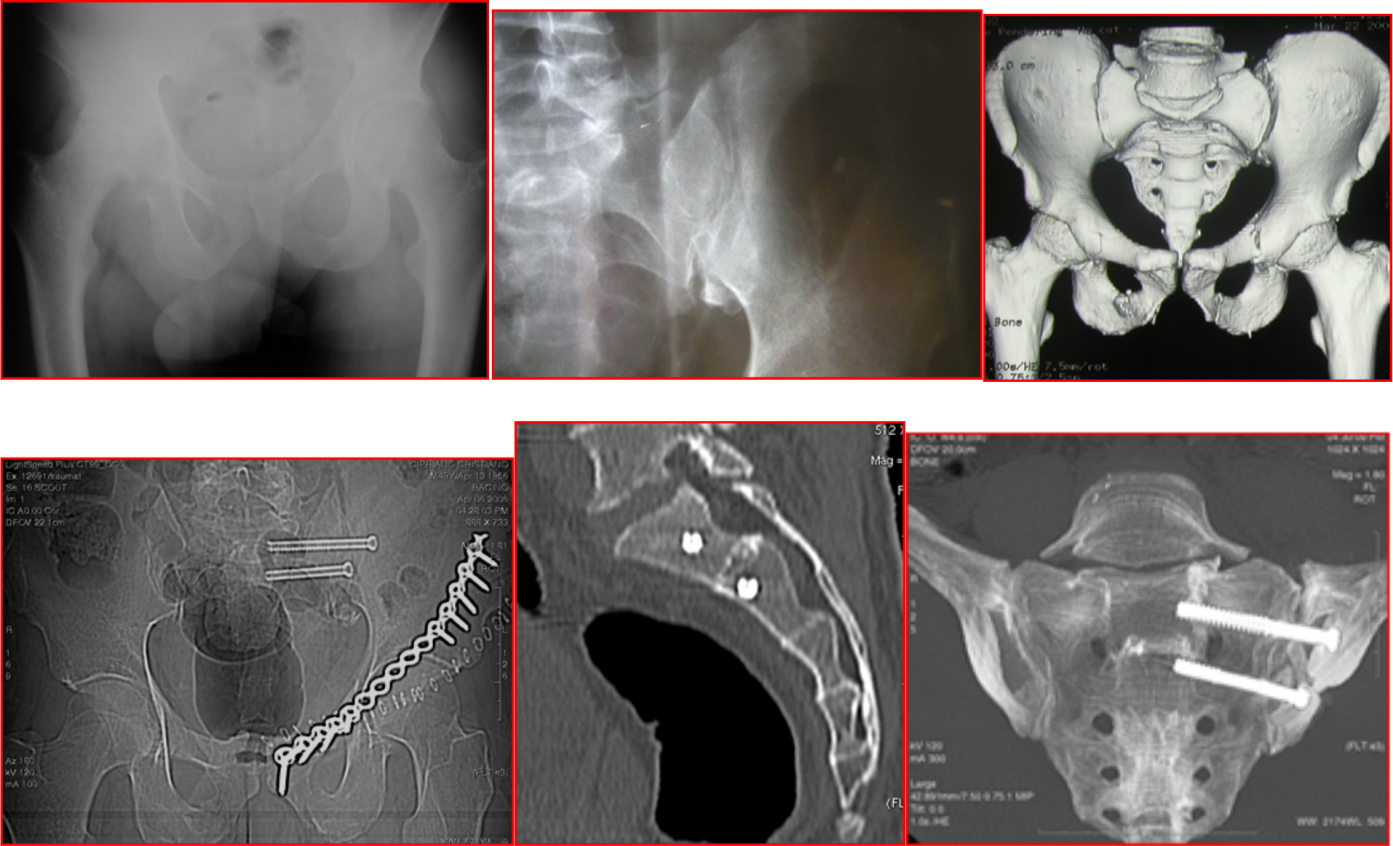
Case of a male patient (49 years old) with C1 Tile fracture, left ileopubic rami fracture and left S-I joint instability following a motorbike accident (top three pictures: AP X-ray view and pelvis 3D CT reconstruction). The patient was initially treated with S-I joint reduction and fracture reduction with plate and screws by anterior approach (bottom-left picture). This was followed (4 days following the initial surgical procedure) by surgical stabilization of the S-I joint with two percutaneous CT-guided cannulated screws within S1 and S2 vertebral body (sagittal and coronal CT-scan views).

**Figure 4:**
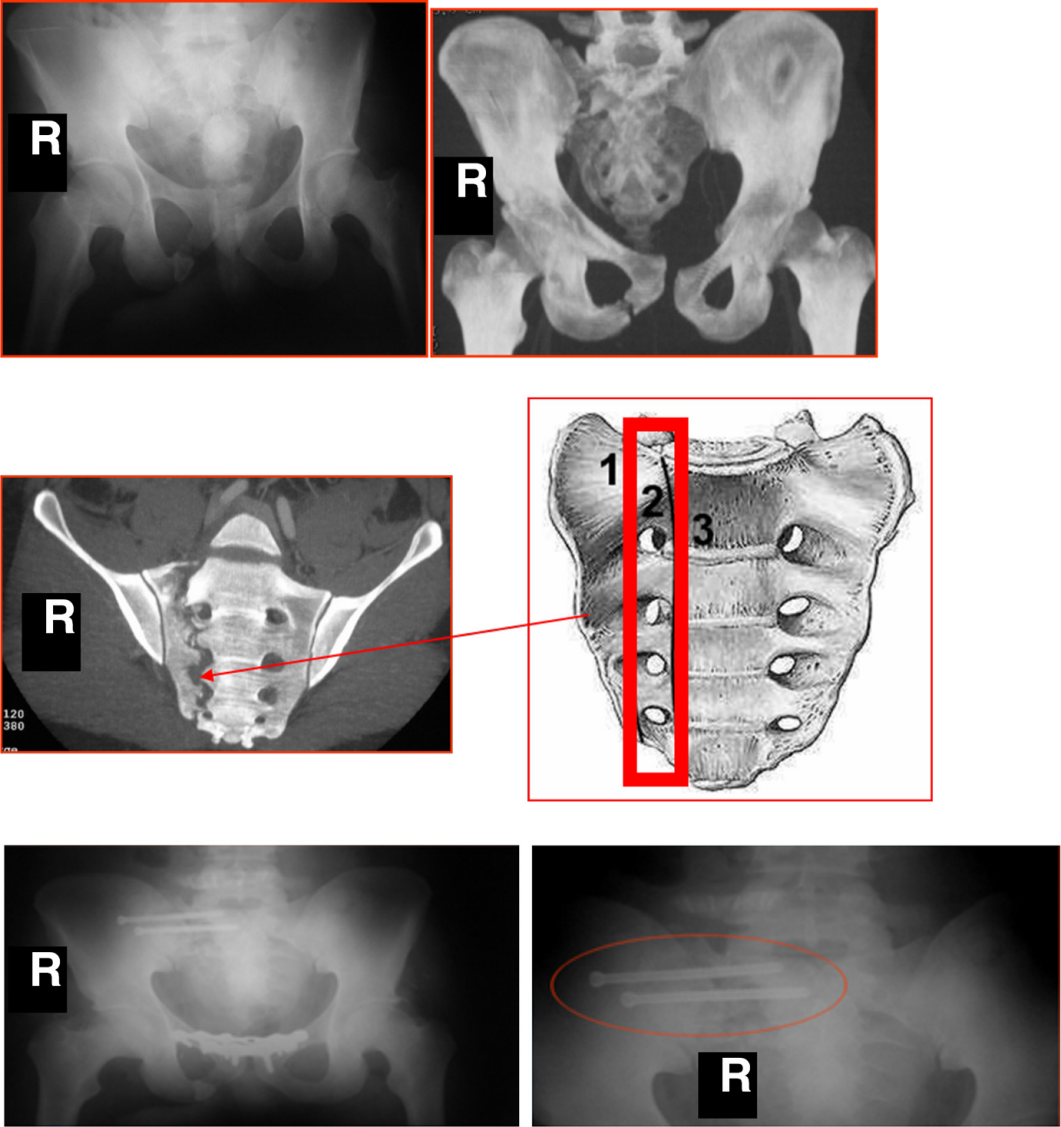
Case of a 19-year-old male patient who sustained a Type 2 Dennis fracture of the sacrum associated with pubic symphysis diastasis and superior subluxation of the right side of the pelvis (type C1) following a fall from height (top pictures: AP radiographic view and pelvis CT-reconstruction; middle pictures: coronal view). The patient was initially treated with reduction and fixation with double plate and screws (anterior stabilization with anterior approach). This was followed by S-I joint fixation with two percutaneous CT-guided screws within S1 and S2 vertebral body 5 days following the initial procedure (bottom pictures: plain radiographs following percutaneous screw fixation).

**Figure 5:**
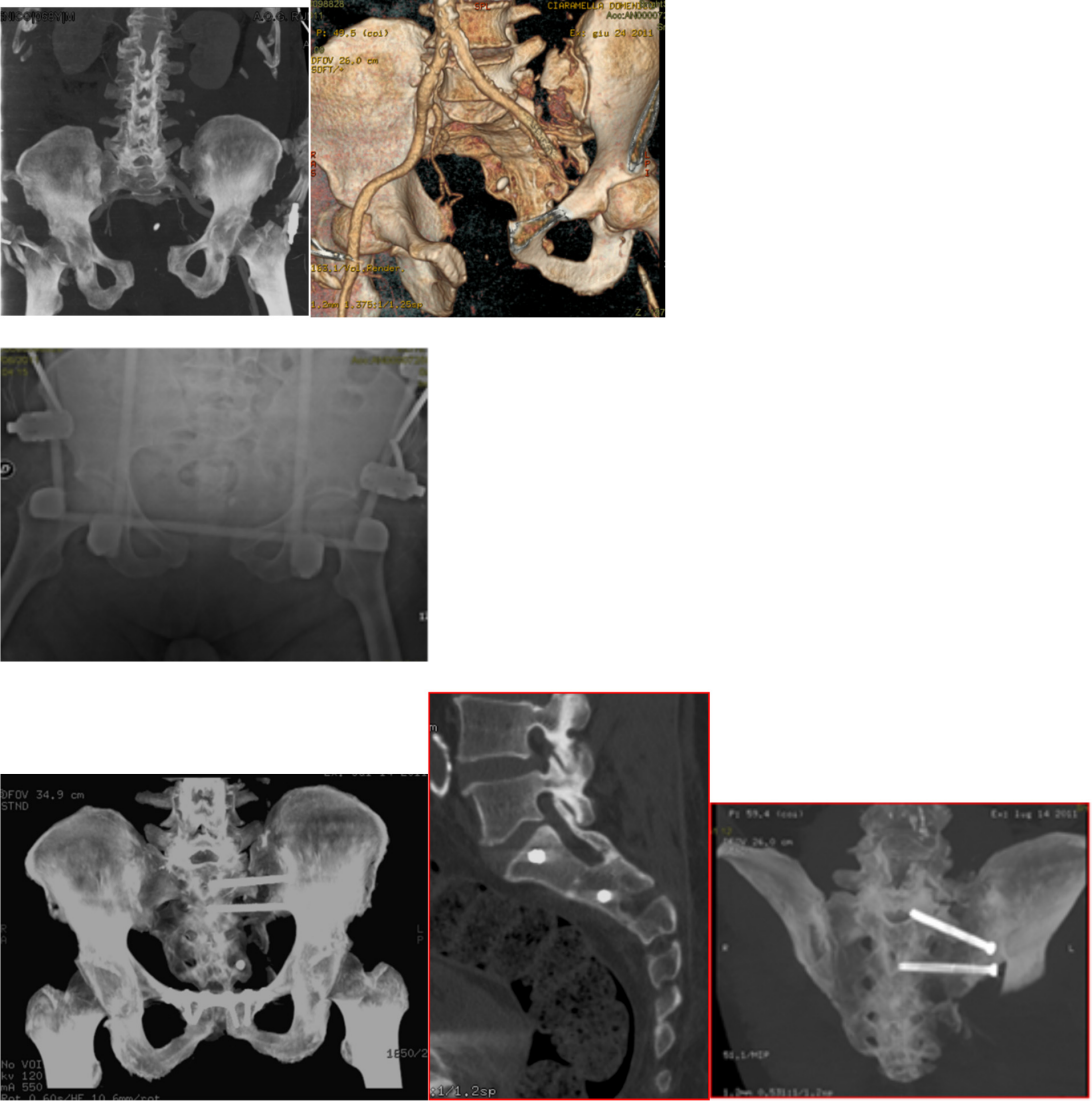
Case of a 55-year-old male patient who sustained a Type III Dennis fracture of the left S-I joint associated with pubic symphysis diastasis (type B1) following a fall from height (top pictures: coronal CT view and CT reconstruction). The injury was treated within few hours from the fall with external fixator (middle pic: AP plain radiograph of the pelvis) and he was then admitted to the ITU ward. This was followed by reduction and fixation with plate and screws 7 days after ITU admission (bottom-left picture). The treatment was completed 3 days after plating with S-I joint reduction and fixation with two percutaneous CT-guided screws within S1 and S2 vertebral body (last two bottom pictures).

Reduction of the sacroiliac instability (Figure 3) or instability of the sacrum (Figures 4 and 5 ) is a tough challenge for the surgeons. The open surgical techniques could give good results in terms of reduction but also have high rate of local and systemic morbidity and are associated with a long learning care [29].

Some authors such as Howlett et al. [30] argue that complications due to stabilization of lesions of the sacrum or sacroiliac by plate fixation decrease with the improved skill of the surgeon and the discovery and use of new innovative techniques. The percutaneous technique is becoming more and more popular for this characteristic of providing tissue sparring for the patient and significant reduction of morbidity/mortality rates [31,32]. Percutaneous techniques, such as synthesis with ileosacral screws, are an interesting treatment option for many authors [8,11–22,31,32]. Routt et al. [8] described that the posterior percutaneous technique (the same used by us) has a low infection rate (0 on a series of 177 consecutive patients). However, this technique (like other percutaneous techniques for the treatment of pelvic fractures) is challenging and requires a perfect knowledge of the anatomy of the pelvis and the indispensable presence of a radiological technician capable of using C-Arm fluoroscopes.

The display brightness may be impossible to be achieved due to severe obesity or intra-abdominal contrast [10,33,34,35]; therefore, CT scans remain the gold standard as an aid for percutaneous stabilization of these lesions [36,37]. The guide wire must be placed in all sacral and sacroiliac fractures with depth and angle based on CT scout scans [18]. As in all surgical pelvic cases, vascular and nerve structures are at high risk of injury; in the percutaneous fracture reduction and fixation, there may develop possible complications such as insufficient reduction, rupture or loosening of the devices, incorrect placement of the screws and neurological lesions.

Routt and Simonian [7] argued that the post-reduction residual gap at different levels must be less than 1 cm. These authors obtained only three malreductions out of 60 sacral fractures treated with closed reduction obtained by manipulation. They also reported five cases of implant failure and two nonunion cases [7,22].

Schweitzer et al. [5] described 71 cases of pelvic ring fractures (10 type B2 and 61 type C), treated with closed reduction and percutaneous screw fixation, obtaining satisfactory reduction in 69 cases and 62 cases of good or excellent functional results, according to the Majeed scoring system [23]. Routt et al. also reported a 9% rate of complication related to the surgical technique [8].

The percutaneous fracture fixation with plate and screws has recently been described [38,39], and its first results are encouraging. This technique, however, has advantages only if the reduction obtained by manual traction is excellent [38,39].

Choy et al. [40] described in their study 32 patients consisting of 21 males and 11 females. The mean age was 41 years (range: 19–76). The average follow-up period was 51 months (range: 36–73). According to the AO-OTA classification, there were 11 cases of B2 lesions, 8 B3 cases, 9 C1 cases, 2 C2 cases and 2 C3 cases. In the posterior lesions, there were 20 cases of sacral fractures and 12 cases of sacroiliac joint disruptions or dislocations. These operations were performed by applying a plate and screws to the anterior pelvic column and subsequent sacroiliac percutaneous fixation. In 16 cases, the clinical results were excellent; 10 cases were good, 4 moderate, 2 cases had poor functional results. Two out of seven cases had poor functional results with residual neurological symptoms. The radiological findings showed 16 cases of anatomical reduction, 9 cases of close anatomical reduction and 7 cases of moderate impairment. All patients had a fracture union, except three cases of nonunion of the pubic branch. The complications encountered were three cases of screw loosening, two cases of rupture of the anterior plaque front and one case of postoperative soft tissue infection. Choy et al. [40] concluded that patients with unstable pelvic ring lesions, stabilized with anterior plate and percutaneous screw stabilization of the sacroiliac joint can be a useful surgical option to recreate the physiological stability of the pelvic ring.

Moed et al. [41] studied 343 patients with fractures of the pelvis with ultrasound monitoring and found out that 35 patients (15%) had asymptomatic deep vein thrombosis: 16 (7%) preoperatively, 19 (8%) postoperative and 2 patients (1%) with negative scans had postoperative symptomatic pulmonary embolism (PE) diagnosed the day after surgery, but neither of them was fatal.

We initially reviewed the published literature and decided to routinely use the studied surgical procedure to treat patients (during the set time frame) with Tile's type C fracture of the pelvis with vertical instability of the SIJ given the good results uniformly achieved internationally. The traditional technique was followed without any subjective change. Good objective results were achieved (assessed by using the Majeed Score, clinical examination, return to work, record of complications and radiological follow-up assessment).

We noticed constant improvement of postoperative functional results through calculation of Majeed scores and related data analysis. These improvements were especially significant during the first post-op year. We felt this was due to the natural physiological characteristic of patients' post-op recovery following major surgery, which is normally quicker in the first weeks from surgery and it gradually slows down till achieving a variable endpoint. We could not justify the second peek of improvement noticed between 2 and 3 years of follow-ups, which was not considered of statistical relevance by our team as it lacked clinical correlation. We attributed the overall good functional results and gradual but constant reduction of the scores to the effectiveness and safety of the studied procedure, which is able to provide a good management option for Tile C fractures with a mini-invasive procedure and good short- and long-term results.

The amount of patients who did not manage to go back to their pre-trauma job is relatively high. However, this has to be taken into account looking at the overall picture of the studied poly-trauma patients. The placement of SI percutaneous screws is a mini-invasive procedure providing satisfactory clinical and radiological results and it does not justify the big impact on the quality of life and function noticed on our patients. Patients with a Tile C injury have usually sustained multiple serious injuries to their anatomical structures (bones, soft tissues, organs, etc.), whose treatment is usually complex and results are variable. We believe that a comparative study with stratification for treatment of different injuries of poly-trauma patients and their results could be carried out in order to confirm our theory and give information with higher level of evidence. Therefore, it is currently hard to scientifically comment on the high rate of jog change recorded, which we however believe it is not significantly affected by the performance of the CT-guided cannulated screw procedure but more probably by the impact of other sustained major injuries.

Considering the severity of the sustained injuries and the invasiveness of the procedures performed to treat them, we could state that the complication rate is relatively low, especially the ones strictly related to the studied procedure with cannulated screws. The need of blood transfusion was never caused by the percutaneous procedure (but by the treatment of the other associated injuries) and no patient needed to be admitted to the intensive care unit. This is also supported by the relatively short length of stay of the patients, with an average of 15 days, and the longest stay of 33 days.

Only two cases of metalwork mobilization were recorded at 16 months post-op which were electively treated successfully with removal of metalwork surgery. This complication has not affected the long-term functional outcomes for these two patients as radiological evidence of fracture consolidation was achieved at about 2 months on average and the metalwork breakage did not significantly affect patients' mobility status nor neurology nor rehabilitation, with the exception of few days of post-op rest and wound care following removal of screws.

We also recorded one case of screw breakage in a psychiatric patient. However, this complication was due to the psychiatric and mental health conditions of the patient and it should not be considered as a real metalwork failure. Therefore, we do not take into account this case in the overall evaluation of our results.

Our results are encouraging and it seems that the use of CT-guided percutaneous cannulated screws to treat the included type of injury should be chosen as management option in absence of specific contraindications. The relatively simple procedure and subsequent smaller operating times, low level of invasiveness, low complication rate, relative short length of stay and good results overall seem to push toward the direction of validating the studied procedure as the most appropriate surgical option in Tile's type C fractures with SIJ vertical instability. This is supported by significant amount of internationally recognized works.

We felt our study had also few limitations. Case series, relatively limited number of patients; non-probability sample of convenience (due to few centric sample, Level 1 Trauma Center); retrospective study (possible presence of confounding factors, possibility to obtain only “associations” but no “causation”, some key statistics cannot be measured); selection of patients may be biased (making generalization of results difficult); use of subjective scores; measurements and intervention were made without randomization of the researcher to the experimental groups; possible presence of temporal confounders. Despite, our study has taken into account a number of cases which could be considered very significant given the severity of the injuries and the complexity of their treatment. Therefore, we believe that our results could be seen as the first step for further validation of the surgical procedure (also considering the already obtained objective good outcomes) possibly with a more powered comparative study.

## Conclusions

Our results suggest that the stabilization of SI Tile type C fracture/dislocations with CT-guided percutaneous cannulated screws is a valid and feasible management option and associated with low complication rates. This is allowed by the low level of invasiveness of the procedure and relatively small operating time. Our data confirm that the surgical percutaneous fixation with cannulated screws of such fractures allows the obtainment of good clinical results both at short and long term, providing early fracture consolidation and good functional outcomes.

We advocate the need of a more powered study and a bigger cohort in order to further validate the appropriateness of the studied surgical procedure and/or unite the inhomogeneous studied groups and results already obtained internationally. A satisfaction questionnaire to be administered to all treated patients could also be useful to assess the results from patients' prospective.

## Funding

None.

## Conflict of interest

All authors disclose any financial and personal relationships with other people or organizations that could inappropriately influence (bias) their work. Examples of potential conflicts of interest include employment, consultancies, stock ownership, honoraria, paid expert testimony, patent applications/registrations, and grants or other funding.

## References

[1] Grubor P, Milicevic S, Biscevic M, Tanjga R (2011) Selection of treatment method for pelvic ring fractures. Med Arh 65 (5), 278–82. 22073851 10.5455/medarh.2011.65.278-282

[2] Tile M (1988) Pelvic ring fractures: should they be fixed? J Bone Joint Surg Br. 70 (1), 1–12. 3276697 10.1302/0301-620X.70B1.3276697

[3] Dalal SA, Burgess AR, Siegel JH, Young JW, Brumback RJ, Poka A, Dunham CM, Gens D, Bathon H (1989) Pelvic fracture in multiple trauma: classification by mechanism is key to pattern of organ injury, resuscitative requirements, and outcome. J Trauma 29 (7), 981–1000. 2746708

[4] Routt ML, Jr, Nork SE, Mills WJ (2000) Percutaneous fixation of pelvic ring disruptions. Clin Orthop Relat Res 375, 15–29. 10.1097/00003086-200006000-00004 10853150

[5] Schweitzer D, Zylberberg A, Córdova M, Gonzalez J (2008) Closed reduction and iliosacral percutaneous fixation of unstable pelvic ring fractures. Injury 39 (8), 869–874. 18621370 10.1016/j.injury.2008.03.024

[6] Nelson DW, Duwelius PJ (1991) CT-guided fixation of sacral fractures and sacroiliac joint disruptions. Radiology 180 (2), 527–532. 2068323 10.1148/radiology.180.2.2068323

[7] Routt ML, Jr, Simonian PT (1996) Closed reduction and percutaneous skeletal fixation of sacral fractures. Clin Orthop Relat Res 329, 121–128. 10.1097/00003086-199608000-00015 8769443

[8] Routt ML, Jr, Simonian PT, Mills WJ (1997) Iliosacral screw fixation: early complications of the percutaneous technique. J Orthop Trauma 11 (8), 584–589. 9415865 10.1097/00005131-199711000-00007

[9] Matta JM, Saucedo T (1989) Internal fixation of pelvic ring fractures. Clin Orthop Relat Res 242, 83–97. 2706863

[10] Tonetti J, van Overschelde J, Sadok B, Vouaillat H, Eid A (2013) Percutaneous ilio-sacral screw insertion: fluoroscopic techniques. Orthop Traumatol Surg Res. 99 (8), 965–972. 24238905 10.1016/j.otsr.2013.08.010

[11] Marsh JL, Slongo TF, Agel J, Broderick JS, Creevey W, DeCoster TA, Prokuski L, Sirkin MS, Ziran B, Henley B, Audigé L (2007) Fracture and dislocation classification compendium −2007: Orthopaedic Trauma Association classification, database and outcomes committee. J Orthop Trauma 21(10 Suppl.), S1–S133. 10.1097/00005131-200711101-00001 18277234

[12] Pape HC, Krettek C (2003) Management of fractures in the severely injured: influence of the principle of “damage control orthopaedic surgery”. Unfallchirurg 106(2), 87–96. 12624681 10.1007/s00113-003-0580-2

[13] Vincent JL, Manikis P (1995) End-points of resuscitation In: The Integrated Approach to Trauma Care. Goris RJA, Trentz O, Editors. Berlin, Springer, pp. 98–105.

[14] Ertel W, Oberholzer A, Platz A, Stocker R, Trentz O (2000) Incidence and clinical pattern of the abdominal compartment syndrome after “damage-control” laparotomy in 311 patients with severe abdominal and/or pelvic trauma. Crit Care Med 28 (6), 1747–1753. 10890613 10.1097/00003246-200006000-00008

[15] Regel G, Lobenhoffer P, Grotz M, Pape HC, Lehmann U, Tscherne H (1995) Treatment results of patients with multiple trauma: an analysis of 3406 cases treated between 1972 and 1991 at a German Level I Trauma Center. J Trauma. 38 (1), 70–78. 7745664 10.1097/00005373-199501000-00020

[16] Bosse MJ, MacKenzie EJ, Riemer BL, Brumback RJ, McCarthy ML, Burgess AR, Gens DR, Yasui Y (1997) Adult respiratory distress syndrome, pneumonia, and mortality following thoracic injury and a femoral fracture treated either with intramedullary nailing with reaming or with a plate. A comparative study. J Bone Joint Surg Am 79 (6), 799–809. 9199375 10.2106/00004623-199706000-00001

[17] Crowl AC, Young JS, Kahler DM, Claridge JA, Chrzanowski DS, Pomphrey M (2000) Occult hypoperfusion is associated with increased morbidity in patients undergoing early femur fracture fixation. J Trauma 48 (2), 260–267. 10697084 10.1097/00005373-200002000-00011

[18] Zhang YZ, Lu S, Xu YQ, Shi JH, Li YB, Feng ZL (2009) Application of navigation template to fixation of sacral fracture using three-dimensional reconstruction and reverse engineering technique. Chin J Traumatol 12 (4), 214–217. 19635214

[19] Ray WA, MacDonald TM, Solomon DH, Graham DJ, Avorn J (2003) COX-2 selective non-steroidal anti-inflammatory drugs and cardiovascular disease. Pharmacoepidemiol Drug Saf 12 (1), 67–70. 12616850 10.1002/pds.798

[20] Tsailas PG, Babis GC, Nikolopoulos K, Soucacos PN, Korres DS (2009) The effectiveness of two COX-2 inhibitors in the prophylaxis against heterotopic new bone formation: an experimental study in rabbits. J Surg Res 151 (1), 108–114. 18656899 10.1016/j.jss.2007.12.804

[21] Vasileiadis GI, Sioutis IC, Mavrogenis AF, Vlasis K, Babis GC, Papagelopoulos PJ (2011) COX-2 inhibitors for the prevention of heterotopic ossification after THA. Orthopedics 34 (6), 467. 21661680 10.3928/01477447-20110427-23

[22] Endo K, Sairyo K, Komatsubara S, Sasa T, Egawa H, Ogawa T, Yonekura D, Murakami R, Yasui N (2005) Cyclooxygenase-2 inhibitor delays fracture healing in rats. Acta Orthop 76 (4), 470–474. 16195060 10.1080/17453670510041439

[23] Majeed SA (1989) Grading the outcome of pelvic fractures. J Bone Joint Surg Br 71(2), 304–306. 2925751 10.1302/0301-620X.71B2.2925751

[24] Buerger PM, Peoples JB, Lemmon GW, McCarthy MC (1993) Risk of pulmonary emboli in patients with pelvic fractures. Am Surg 59, 505–508. 8338280

[25] Norwood SH, McAuley CE, Berne JD, Vallina VL, Kerns DB, Grahm TW, McLarty JW (2001) A potentially expanded role for enoxaparin in preventing venous thromboembolism in high risk blunt trauma patients. J Am Coll Surg 192 (2), 161–167. 11220715 10.1016/s1072-7515(00)00802-4

[26] Meissner MH, Chandler WL, Elliott JS (2003) Venous thromboembolism in trauma: a local manifestation of systemic hypercoagulability? J Trauma 54, 224–231. 12579044 10.1097/01.TA.0000046253.33495.70

[27] Cipolle MD, Wojcik R, Seislove E, Wasser TE, Pasquale MD (2002) The role of surveillance duplex scanning in preventing venous thromboembolism in trauma patients. J Trauma 52, 453–462. 11901319 10.1097/00005373-200203000-00007

[28] Stannard JP, Singhania AK, Lopez-Ben RR, Anderson ER, Farris RC, Volgas DA, McGwin GR, Jr, Alonso JE (2005) Deep-vein thrombosis in high-energy skeletal trauma despite thromboprophylaxis. J Bone Joint Surg Br 87 (7), 965–968. 15972912 10.1302/0301-620X.87B7.15989

[29] Matta JM, Tornetta P, III (1996) Internal fixation of unstable pelvic ring injuries. Clin Orthop Relat Res 329, 129–140. 10.1097/00003086-199608000-00016 8769444

[30] Howlett A, Nork SE, Routt M (2005) Perioperative complications after open posterior pelvic surgery, in: Proceedings of the 2005 Annual Meeting of American Academy of Orthopaedic Surgeons, Resemont IL, p. 602.

[31] Nicodemo A, Cuocolo C, Capella M, Deregibus M, Massè A (2011) Minimally invasive reduction of vertically displaced sacral fracture without use of traction table. J Orthop Traumatol 12 (1), 49–55. 21347808 10.1007/s10195-011-0132-4 PMC3052429

[32] Quintero AJ, Tarkin IS, Pape HC (2009) Case report. The prone reduction of a sacroiliac disruption with a pelvic C-clamp. Clin Orthop Relat Res 467 (4), 1103–1106. 18810568 10.1007/s11999-008-0508-9 PMC2650064

[33] Wolinsky P, Lee M (2007) The effect of C-arm malrotation on iliosacral screw placement. J Orthop Trauma 21 (7), 427–434. 17762471 10.1097/BOT.0b013e318137948d

[34] Peng KT, Huang KC, Chen MC, Li YY, Hsu RW (2006) Percutaneous placement of iliosacral screws for unstable pelvic ring injuries: comparison between one and two C-arm fluoroscopic techniques. J Trauma 60 (3), 602–608. 16531861 10.1097/01.ta.0000200860.01931.9a

[35] Ray WZ, Ravindra VM, Schmidt MH, Dailey AT (2013) Stereotactic navigation with the O-arm for placement of S-2 alar iliac screws in pelvic lumbar fixation. J Neurosurg Spine 18 (5), 490–495. 23495892 10.3171/2013.2.SPINE12813

[36] Džupa V, Krbec M, Kadeřábek R, Rusnák R, Douša P, Skála-Rosenbaum J, Fridrich F, Báča V, Grill R (2013) Intraoperative CT navigation in spinal and pelvic surgery: initial experience. Rozhl Chir 92 (7), 379–384. 24003877

[37] Zheng Z, Zhang Y, Hou Z, Hao J, Zhai F, Su Y, Pan J (2012) The application of a computer-assisted thermoplastic membrane navigation system in screw fixation of the sacroiliac joint: a clinical study. Injury 43 (4), 495–499. 22284333 10.1016/j.injury.2011.12.022

[38] Dolati B, Larndorfer R, Krappinger D, Rosenberger RE (2007) Stabilization of the posterior pelvic ring with a slide-insertion plate. Oper Orthop Traumatol 19 (1), 16–31. 17345025 10.1007/s00064-007-1193-7

[39] Krappinger D, Larndorfer R, Struve P, Rosenberger R, Arora R, Blauth M (2007) Minimally invasive transiliac plate osteosynthesis for type C injuries of the pelvic ring: a clinical and radiological follow-up. J Orthop Trauma 21 (9), 595–602. 17921833 10.1097/BOT.0b013e318158abcf

[40] Choy WS, Kim KJ, Lee SK, Park HJ (2012) Anterior pelvic plating and sacroiliac joint fixation in unstable pelvic ring injuries. Yonsei Med J 53 (2), 422–426. 22318833 10.3349/ymj.2012.53.2.422 PMC3282962

[41] Moed BR, Miller JR, Tabaie SA (2012) Sequential duplex ultrasound screening for proximal deep venous thrombosis in asymptomatic patients with acetabular and pelvic fractures treated operatively. J Trauma Acute Care Surg 72 (2), 443–447. 22327985 10.1097/TA.0b013e318241090d

